# Relationship Between Clinical Course of Treatment for Burning Mouth Syndrome in Early Period and Aggression Using Rosenzweig Picture Frustration Study: A Retrospective Observational Study

**DOI:** 10.7759/cureus.60174

**Published:** 2024-05-13

**Authors:** Yojiro Umezaki, Rui Egashira, Haruhiko Motomura, Trang T Tu, Toru Naito

**Affiliations:** 1 Department of Geriatric Dentistry, Fukuoka Dental College, Fukuoka, JPN; 2 Department of Basic Dental Sciences, Faculty of Odonto-Stomatology, University of Medicine and Pharmacy at Ho Chi Minh City, Ho Chi Minh, VNM

**Keywords:** retrospective study, aggression, improvement course, rosenzweig picture frustration study, burning mouth syndrome

## Abstract

Objective: Burning mouth syndrome (BMS) is characterized by a chronic, ongoing sensation of intraoral burning or discomfort without causative lesions. This study sought to examine the relationship between personality traits in patients with BMS using the Rosenzweig Picture Frustration (PF) study, a projective psychological test, and their progress in treatment.

Methods: Data were collected from outpatients diagnosed with BMS at our clinic between April 2017 and March 2021. The data were analyzed for 28 patients with BMS, of which nine showed improvement earlier than three months (early responders; ER), and the others did not (non-early responders; NER).

Results: The mean visual analog scale (VAS scores for BMS pain at the first visit were 52.8 in the ER and 59.6 in NER (n.s.). No significant differences were detected in the type and direction of aggression between ER and NER in the PF study. In contrast, the group conformity score of the ER (63.7%) was significantly higher than that of the NER (51.4%).

Conclusions: Personal traits reflected in the PF study may have affected the course of improvement in the BMS. To understand the characteristics of patients with BMS and achieve more favorable treatment outcomes, further study on their personality organization is necessary.

## Introduction

Burning mouth syndrome (BMS) is classified as an idiopathic orofacial pain in the International Classification of Orofacial Pain [1], of which symptom is chronic intraoral burning or unpleasant sensation without any causative lesions. It can be accompanied by other sensory disorders, such as xerostomia [2] and taste disturbance [2,3] with or without hyposalivation. In typical cases, burning pain worsens during the day but is alleviated while eating or concentrating on anything but the pain itself [4]. A recent estimation showed the worldwide prevalence of BMS is 1.73% in a population-based study [5]. Women develop BMS more frequently, especially those in the post-menopausal stage and between 50 and 70 years of age [6]. BMS is commonly said to impact the quality of life significantly [7,8]. BMS is considered poorly responsive to current therapies, with less than half of patients showing improved symptomatology [9]. However, many studies have reported relatively better outcomes with antidepressants [10-13].

Although the cumulative outcome of antidepressants for BMS is favorable, the course of improvement of BMS is variable. Whereas some patients respond quickly to treatment, it is more challenging to treat others. Moreover, simply providing a diagnosis and explanation of the pathology of BMS can reduce patient anxiety, resulting in pain relief in some cases. These individual differences are believed to be related to each patient's personality, intellectual ability, and psychosocial background. We recently reported that the personality traits were different between BMS patients with and without a history of depression [14]; however, the relation of personality to treatment outcome remains unclear. This study analyzed personality traits in patients with BMS using the Rosenzweig Picture Frustration (PF) study, a projective psychological test, and then examined its relationship with the improvement course.

## Materials and methods

Participants

Data were collected from outpatients diagnosed with BMS at our clinic between April 2017 and March 2021. The diagnosis of BMS was made by a clinician certified by the Japanese Society of Psychosomatic Dentistry, based on the International Classification of Headache Disorder third edition (ICHD-3) criteria [15] and medical interviews and intra or extraoral findings at the first visit to our clinic. A PF study was conducted for 30 patients who consented to the study in the pool of 293 patients with primary BMS. Two patients were excluded from the analysis to calibrate data. The reasons for this were male sex (n=1) and comorbid diagnosis of oral cenesthopathy (n=1).

Treatment setting

At the first visit to our clinic after the diagnosis of BMS, we explained the disease, treatment methods, and drug therapies and predicted the course of treatment. Additionally, we explained that there were many patients with similar symptoms and that many patients had improved.

**Figure 1:**
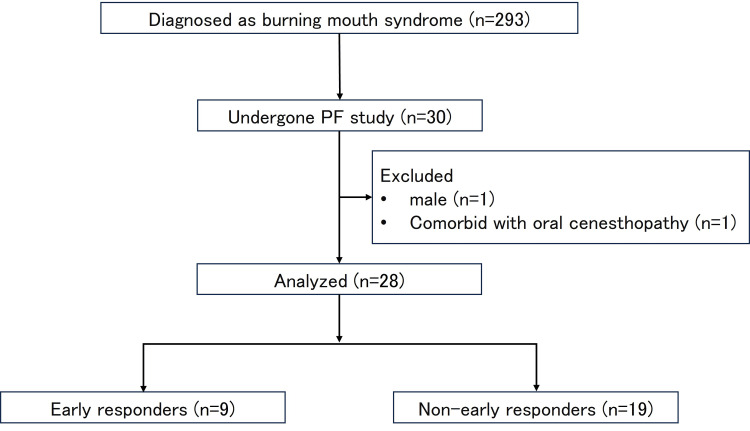
Flowchart of data collection A Rosenzweig Picture Frustration (PF) study was conducted in 30 selected patients with burning mouth syndrome. Finally, data from 28 patients with BMSs were analyzed. Of the 28 patients with BMS, nine were allocated to the early responders (ER) group, and 19 were assigned to the non-early responders (NER) group.

Antidepressants have been effective treatment options for BMS [16]. If the patients agreed to treatment, we also administered the medication after a detailed explanation. The effects and adverse events were examined a week after the first dose, and the dosage was adjusted constantly by two or three weeks. Even if the patients disagreed with the medication, periodic observations and brief lifestyle education were continued. Subjective pain intensity was assessed at each visit using a visual analog scale (VAS) ranging from 0 (no pain) to 100 (very severe pain). Outcomes three months after the first visit were evaluated using Clinical Global Impressions (CGI; [17]). The CGI was assessed by a clinician, who diagnosed the patients with BMS, as follows: 1, very much improved; 2, much improved; 3, minimally improved; 4, no change; 5, minimally worse; 6, much worse; and 7, very much worse. In this study, patients with a CGI score of 1 (very much improved) were assigned to the favorable progress group in the early phase (early responders; ER), and patients with a CGI score other than 1 were assigned to the unfavorable progress group in the early phase (non-early responders; NER). In the present study, nine and 19 patients were assigned to the ER and NER groups, respectively. A flowchart of the data collection in this study is shown in Figure 1.

PF study

The adult form of the PF study was conducted during treatment to understand patient features. As is commonly recognized, the PF study comprises a test leaflet with 24 sketches of frustrating situations. A verbal utterance by one of two or more individuals was printed on a tag to cause frustration in the test participant. The test participants have the task of writing down the response that they believe the frustrated person could give in the empty speech flags.

According to Rosenzweig [18,19], frustration comprises either a so-called “ego blockage” or a “superego blockage.” Ego-blocking situations (EBS) occur when an obstacle - personal or impersonal - inhibits, disappoints, hinders, or otherwise impairs the participant’s expansiveness. Adult forms include situations 1, 3, 4, 6, 8, 9, 11, 12, 13, 14, 15, 18, 20, 22, 23, and 24. Conversely, Rosenzweig describes situations where the participant is blamed, accused, or reprimanded by another person as superego-blocking situations (SBS). In the adult form, these were situations 2, 5, 7, 10, 16, 17, 19, and 21 [18,19].

Each response is scored for its expressed direction of aggression (extra-punitiveness, intro-punitiveness, and im-punitiveness) and type of reaction (an obstacle-dominant, an ego-defensive, and a need-persisted). The scoring components of the PF study are E’ (extrapeditive), E (Extrapunitive), E_ (a variant of E), e (extrapersistive), I’ (Intropeditive), I (Intropunitive), I_ (a variant of I), I (Intropersistive), M’ (impeditive), M (Impunitive) and m (Impersistive). The sum scores of extra-punitiveness (Cat. E), intro-punitiveness (Cat. I), im-punitiveness (Cat. M) and obstacle-dominant (O-D), ego-defensive (E-D), and need-positive (N-P) were also evaluated (Table 1). In this study, two clinicians majoring in psychiatry and psychosomatic dentistry evaluated the patient responses. If the evaluation was split between the two clinicians, the final score was determined based on the discussion.

**Table 1:** Components of PF study The scoring components of PF study are E’ (extrapeditive), E (Extrapunitive), E_ (a variant of E), e (extrapersistive), I’ (Intropeditive), I (Intropunitive), I_ (a variant of I), I (Intropersistive), M’ (impeditive), M (Impunitive), and m (Impersistive). The sum scores of extra-punitiveness (Cat. E), intro-punitiveness (Cat. I), im-punitiveness (Cat. M), obstacle-dominant (O-D), ego-defensive (E-D), and need-persistive (N-P) were also evaluated.

		Type of aggression		
		Obstacle-Dominance (O-D)	Ego-Defense (E-D)	Need-Persistence (N-P)
DIRECTION OF AGGRESSION	extra-punitiveness (Cat. E)	E’ (extrapeditive)	E (Extrapunitive) E_ (a variant of E)	e (extrapersistive)
	intro- punitiveness (Cat. I)	I’ (Intropeditive)	I (Intropunitive) I_ (a variant of I)	I (Intropersistive)
	im- punitiveness (Cat. M)	M’ (impeditive)	M (Impunitive)	m (Impersistive)

Group Conformity Rating (GCR) scores were computed as the rate of concordance (%) with the normal population. This score corresponded to the percentage of popular responses (%P) in the Rorschach test. The GCR score was obtained by comparing the participants’ scores with those expected for 18 items (2, 3, 4, 5, 6, 7, 10, 11, 13, 15, 16, 17, 18, 19, 21, 22, 23, and 24). In particular, those previously found to produce a specific variety of responses significantly often justify their use as criteria. The mean GCR scores of elderly Japanese females and males are 52.5% and 52.8% [20]. This discrepancy between females and males is a reason for the exclusion of male patients mentioned above.

Statistical analysis

All data, including sex, age, results of the PF study, and treatment outcomes, were collected retrospectively. Data were analyzed using the Mann-Whitney U test, Spearman’s correlation coefficient and chi-square test using SPSS Windows software version 28.0 (IBM Corp., Armonk, NY). Statistical significance was set at P < 0.05. This study was approved by the Ethics Committee of Fukuoka Dental College (approval no. 503).

## Results

Table 2 presents the demographic data of the participants in the ER and NER groups. The mean age of 28 collected patients was 68.5 years old (SD 11.0). Further, the duration of illness at the first visit was 41.4 months (SD 72.5). The ER group comprised nine individuals with a mean age of 64.2 years old (SD 12.7) and a mean duration of illness of 14.6 months (SD12.7). The NER group included 19 individuals with a mean age of 70.5 years old (SD 9.9) and a mean duration of illness of 54.1 months (SD 85.3). The mean age and illness duration were not significantly different between the ER and NER groups. Four patients and a patient had a history of psychiatric disorders in ER and NER, respectively. Two ER patients had a history of depression, which was in the remission phase. Two patients in the ER and one in the NER had a history of anxiety disorder. Various histories of physical conditions such as hypertension, hyperlipidemia, heart disease, and gastroesophageal reflux disease were detected. Nonetheless, there were no significant differences between the ER and NER groups. One patient in the ER and two patients in the NER did not take any medication for BMS, but the others were treated with antidepressants, including amitriptyline, mirtazapine, and aripiprazole.

**Table 2:** Demographic data for early responders and non-early responders ER, early responders; NER, non-early responders; VAS, visual analog scale

	ER	NER	P-value
Female/male	9/0	19/0	n.s.
Age (years-old)	64.2 ± 12.7	70.5 ± 9.9	n.s.
Duration of illness (months)	14.6 ± 12.7	54.1 ± 85.3	n.s.
Initial VAS score	52.8 ± 33.4	59.6 ± 22.8	n.s.

Mean VAS scores for BMS pain at the first visit were 52.8 (SD 33.4) in ER and 59.6 (SD 22.8) in NER (no significant difference). The mean VAS scores at three months after the first visit were 19.3 (SD 29.0) in the ER and 34.3 (SD 25.3) in the NER group (no significant difference). A two-way ANOVA showed no significant differences between the two groups.

In a comparison of the analysis of the PF study between two groups, no apparent significant difference is discerned for the appearance of factors E’, E, E_, e, I’, I, I_, I, M’. M, m, Cat. E, Cat. I, Cat. M, O-D, E-D, and N-P in the ER and NER groups. Specifically, no specific features of the direction and type of aggression were detected between the ER and NER. However, the GCR score differed significantly between the ER (63.7% (SD 11.2)) and NER (51.4% (SD 9.5)) groups (p = 0.009). Additionally, the GCR scores in the SBS were 68.9% (SD 11.7) in the ER group and 64.1% (SD 16.7) in the NER group (no significant difference). In contrast, the GCR scores in the EBS were significantly different between the ER (59.7% (SD 14.8)) and NER (41.8% (SD 9.1)) groups (p = 0.002) (Table 3).

**Table 3:** Appearance numbers and rates of components of Picture Frustration study in early and non-early responder groups ER; early responders, NER; non-early responders, E’; extrapeditive, E; Extrapunitive, E_; a variant of E, e; extrapersistive, I’; Intropeditive, I; Intropunitive, I_; a variant of I, I; Intropersistive, M’; impeditive, M; Impunitive, m; Impersistive, Cat. E: Extra-punitiveness; Cat. I: intro-punitiveness; Cat. M; im-punitiveness, O-D; obstacle-dominant, E-D; ego-defensive, N-P; need-persistive, GCR; Group Conformity Rating, SBS; super-ego blocking situation, EBS; ego-blocking situation.

	ER	NER	P-value
E'	2.56 ± 1.33	1.68 ± 1.33	n.s.
E, E_	1.83 ± 2.09	3.39 ± 2.23	n.s.
e	1.72 ± 1.57	2.37 ± 1.53	n.s.
I'	1.78 ± 1.38	1.58 ± 1.37	n.s.
I, I_	4.28 ± 1.30	4.16 ± 1.42	n.s.
i	2.28 ± 1.44	2.42 ± 1.46	n.s.
M'	2.44 ± 1.36	2.05 ± 1.36	n.s.
M	4.61 ± 1.73	3.84 ± 1.68	n.s.
m	1.72 ± 1.10	1.66 ± 1.05	n.s.
Cat. E	6.11 ± 2.36	7.45 ± 2.54	n.s.
Cat. I	8.33 ± 0.97	8.16 ± 2.15	n.s.
Cat. M	8.78 ± 1.84	7.55 ± 1.70	n.s.
O-D	6.78 ± 2.12	5.32 ± 2.24	n.s.
E-D	10.72 ± 1.35	11.39 ± 2.41	n.s.
N-P	5.72 ± 2.09	6.45 ± 2.77	n.s.
GCR (%)	63.69 ± 11.20	51.44 ± 9.54	p = 0.009
GCR in SBS (%)	68.89 ± 11.70	64.12 ± 16.70	n.s.
GCR in EBS (%)	59.72 ± 14.80	41.82 ± 9.05	p = 0.002

## Discussion

Our findings are as follows: (1) there was no difference in personality traits between the ER and NER using the PF study, and (2) the GCR of the ER was significantly higher than that of others. This is the first report about personality traits related to early responsiveness for treatment in the patients with BMS.

The Picture-Frustration Study, developed by Saul Rosenzweig, is a test used to grasp a person's personality by presenting an illustration of a frustrating situation to the examinee and analyzing their response. The test comprised 24 frustration situations (16 EBS and eight SBS). The frustration responses in the test were categorized and analyzed using nine factors. These were based on combinations of the three directions of aggression (Cat. E, Cat. I, and Cat. M) and three types of aggression (OD, E-D, and N-P). In this study, no specific differences in personality traits between the better outcome group and the others were detected regarding the direction and types of aggression or the nine factors. However, the GCR of the better outcome group was significantly higher than that of the other groups.

Although specific personality traits that influence the treatment outcome are critical in clinical psychology and psychiatry, no unified view has been reached [21]. Many studies have been conducted on the association between the level of personality organization (PO) and psychotherapy responses. A systematic review by Koelen et al. [22] suggested that higher initial PO levels are moderate to strongly associated with better treatment outcomes. Assessments of PO, mainly using interviews, projective techniques, or semi-structured interviews, include the Social Cognition and Object Relations Scale, the Rorschach test, the Inventory of Personality Organization, and part of the MMPI. Most measures of PO evaluate the severity of the personality pathology dimension, which fundamentally differs from representative personality traits such as the Big Five. PO is a latent construct that can only be inferred from manifest indicators, such as identity integration, diffusion, primitive and mature defense mechanisms, reality testing, quality of object relations, moral functioning, and personality rigidity. Thus, The PO level may, without fear of misinterpretation, reflect stages of personality development in both adaptive and maladaptive personality persons. A PF study, in which defense mechanisms are closely related to the underlying theory of the test, may measure part of the level of PO.

In particular, the GCR in the test was developed as a measure that reflects the degree to which a participant's responses agree with those usually given by a normative group [23]. Among the 24 scenes in the test, situations in which a particular type of scored response was relatively high were selected, and their one of the direction and type of aggression were determined to be standard. The degree of coincidence between the participants and standard responses was calculated as the GCR. The higher GCR score means “healthier in mental status” and “more popular responses among the public.” The surrounding individuals may accept a high GCR score. Specifically, a person with a high score may have learned and acquired the reactions that a belonging society expects from them. Thus, the GCR scores, an indicator of social conformity, may also be closely related to the level of PO. The fact that the GCR scores in the ER were higher than those in others suggests that patients with BMS in the ER clearly understand the explanations for the disease and treatment of BMS. Even in interactions that take place in clinical situations, such as explaining the disease, explaining treatment methods and drug therapies, and predicting the course of treatment, people who respond in a standard way may differ from those who do not in the way they understand the explanation and become anxious about the treatment. For example, if a person is highly distrustful of everything and feels victimized, they may not immediately trust explanations of the disease and prospects for treatment given by the therapist. They may be more hesitant about drug therapy. BMS symptoms that general dentists cannot handle often require time to reach a specialist. As a result, anxiety about the disease intensified. A high GCR score may accept explanations for the disease and treatment with open arms, resulting in the reduction of anxiety. In this study, the three-month outcome might reflect, at least in part, the reactivity of psychological factors.

Interestingly, the differences in GCR in the EBS were more prominent than those in the SBS. EBS are those in which some obstacles either personal or impersonal, interrupt, disappoint, deprive, or otherwise directly frustrate the subject [18]. Essentially, the participants were not at fault. Conversely, SBS represents the accusation, charge, or incrimination of the participant by someone else [18]. This is because of the apparent participant’s fault. SBS concerns feelings of guilt or remorse caused by violating rules and unethical or immoral behaviors. Rauchfleisch [24] examined the reaction differences in both EBS and SBS between mentally healthy participants and neurotics. The findings indicate that mentally healthy individuals and neurotics differ markedly in their responses to EBS and SBS, and significant differences can be found in the EBS in many items. Fewer differences can be found in the reactions to SBS between mentally healthy individuals and neurotics. This result may indicate that mentally vulnerable individuals are more likely than healthy individuals to exhibit various responses to EBS but not to SBS. Altogether, the present result that patients with BMS in the ER showed higher GCR scores in EBS not in SBS suggests that relatively mentally healthy patients might tend to improve earlier.

Many patients’ symptoms improved to some degree simply by being told that many patients had similar symptoms when they saw a specialist. Thus, this indicates that the anxiety and fear associated with the original BMS symptoms cannot be ignored. BMS pain is reported to be reduced by a placebo in some systematic reviews [25,26]. In this review, the keys to treatment success are explained. They include correct diagnosis, patient’s acceptance, patient’s understanding of the likely clinical course, patient's participation in the elaboration of a treatment strategy, compliance, positive feedback during treatment, and the ongoing interest of clinicians. Building trust and reassurance among patients is essential for BMS management. Understanding features of aggression or other PO in patients is vital for clinicians specializing in oral psychosomatic disorders, including BMS.

As a limitation, the number of participants in this study was small. Future replications with larger participants are required, which would generalize our findings.

## Conclusions

The GCR scores in the PF study of patients with BMS with better outcomes were higher than the others. However, the appearances of each component of the direction and type of aggression were not different. Patients with high GCR scores may accept explanations for the disease and treatment with open arms, resulting in the early improvement of BMS symptoms. Further study of PO in patients will be necessary to understand the features of patients with BMS and obtain better treatment outcomes.
